# Repeated Application of Autologous Bone Marrow-Derived Lineage-Negative Stem/Progenitor Cells—Focus on Immunological Pathways in Patients with ALS

**DOI:** 10.3390/cells9081822

**Published:** 2020-08-01

**Authors:** Bartłomiej Baumert, Anna Sobuś, Monika Gołąb-Janowska, Edyta Paczkowska, Karolina Łuczkowska, Dorota Rogińska, Alicja Zawiślak, Sławomir Milczarek, Bogumiła Osękowska, Wioletta Pawlukowska, Agnieszka Meller, Karolina Machowska-Sempruch, Agnieszka Wełnicka, Krzysztof Safranow, Przemysław Nowacki, Bogusław Machaliński

**Affiliations:** 1 Department of General Pathology, Pomeranian Medical University, 70-111 Szczecin, Poland; bbaumert@pum.edu.pl (B.B.); ania.sobus@gmail.com (A.S.); edyta.paczkowska@pum.edu.pl (E.P.); karolinaluczkowska58@gmail.com (K.Ł.); doroginska@gmail.com (D.R.); alicja.zawislak@pum.edu.pl (A.Z.); slawek.milczarek@gmail.com (S.M.); bogumilaosekowska@gmail.com (B.O.); 2 Department of Neurology, Pomeranian Medical University, 71-252 Szczecin, Poland; monikagj@op.pl (M.G.-J.); wpawluko@pum.edu.pl (W.P.); agoschorska@gmail.com (A.M.); karolinamachowska88@gmail.com (K.M.-S.); awelnicka@gmail.com (A.W.); przemyslaw.nowacki@pum.edu.pl (P.N.); 3 Department of Medical Rehabilitation and Clinical Physiotherapy, Pomeranian Medical University, 71-210 Szczecin, Poland; 4 Department of Biochemistry and Medical Chemistry, Pomeranian Medical University, 70-111 Szczecin, Poland; chrissaf@mp.pl

**Keywords:** amyotrophic lateral sclerosis (ALS), lineage-negative cells, immunological pathways, inflammatory response, neurotrophins, gene microarrays, miRNA

## Abstract

Therapeutic interventions in amyotrophic lateral sclerosis (ALS) are still far from satisfying. Immune modulating procedures raise hopes for slowing the disease progression. Stem cell therapies are believed to possess the ability to regulate innate and adaptive immune response and inflammation processes. Hence, three intrathecal administrations of autologous bone marrow-derived lineage-negative (Lin^–^) cells were performed every six weeks in 40 sporadic ALS patients. The concentrations of inflammatory-related proteins and expression profiles of selected miRNA in the cerebrospinal fluid (CSF) and plasma at different timepoints post-transplantation were quantified by multiplex Luminex and qRT-PCR. The global gene expression in nucleated blood cells was assessed using the gene microarray technique. According to the ALS Functional Rating Scale (FRSr), the study population was divided into responders (group I, n = 17) and non-responders (group II, n = 23). A thorough analysis of the pro-inflammatory expression profiles, regulated miRNA pathways, and global gene expression profiles at the RNA level revealed the local and systemic effects of Lin^–^ cell therapy on the immune system of patients with ALS. The autologous application of Lin^–^ cells in CSF modulates immune processes and might prevent the progression of neurodegeneration. However, further in-depth studies are necessary to confirm the findings, and prolonged intervention is needed to maintain therapeutic effects.

## 1. Introduction

Immunological processes are known to be implicated in the pathogenesis of multiple neurodegenerative diseases. Emerging evidence indicates the involvement of immunological pathways in the progression of Alzheimer’s disease, multiple sclerosis, epilepsy, Parkinson’s disease, and amyotrophic lateral sclerosis (ALS) [1]. ALS is a lethal, progressive, neurodegenerative disease that affects the lower and upper motor neurons, leading to the gradual weakening of muscles and death, usually within 3–5 years after the onset of the first symptoms [2]. The only available treatment options are riluzole, which inhibits glutamate release and prolongs survival by 2–3 months, and edaravone (approved for use in ALS patients only in Japan, USA, Canada, South Korea, Switzerland, and China), delaying the course of the disease in a subgroup of patients with an early diagnosis and relatively mild phenotype (constituting about 7% of all patients) [3]. Only about 5–10% of cases are familial, while the remaining 90–95% occur sporadically (sALS). The pathogenesis of ALS remains not entirely elucidated. New treatment strategies based on the disease pathomechanism are being developed. The most important factors contributing to the loss of motor neurons described until now are increased excitotoxicity resulting from the impaired uptake of glutamate, a dysfunctional blood-brain barrier (BBB), oxidative stress, mitochondrial dysfunction, the accumulation of misfolded proteins, and inflammation on both a local and systemic level [4,5].

Despite the fact that ALS lacks the characteristics of autoimmune disease, there is increasing evidence proving that various factors responsible for inflammatory processes are increased not only in the central nervous system (CNS), manifested as microglial activation, but also in the tissues and blood of ALS patients [6,7]. There is evidence of immune cell (macrophages, mast cells, and T lymphocytes) infiltration and complement element accumulation in the area of degenerating motor neurons [8,9]. Moreover, the peripheral immune system is also dysregulated, which is reflected in an increased total count of lymphocytes, granulocytes, and neutrophils in the blood of ALS patients [10,11]. It has been shown that those changes are correlated with disease progression [11]. More evidence for the importance of immune response role in disease progression can be found in studies focused on C-reactive protein (CRP) and tumor necrosis factor α (TNF-α) assessment in ALS patients. It has been reported that the level of these proteins is increased in the course of the disease and correlates positively with disease progression [12,13]. Therefore, several attempts have been made to alter the activation of immune response in ALS patients; however, to date none of them have demonstrated beneficial outcomes [14,15,16]. Other circulating molecules which are under investigation in relation to the progression of ALS are miRNAs—small, non-coding oligonucleotides which affect gene expression. In particular, those involved in the regulation of genes related to immune processes and myogenesis (so called “immune-miRs” and “myo-miRs”) seem to pose an interesting object of research in ALS studies. It has been already reported that some of those molecules correlate with the course of the disease [17].

Due to the lack of availability of effective pharmacological treatment options, in recent years there have been multiple approaches utilizing stem cell therapies in an attempt to slow down disease progression. Emphasis in those trials is put mostly on the trophic support, which can be provided via cells’ application to the environment of degenerating neurons [5]. Stem cell application could potentially exert positive effects via the release of neurotrophins (NTs) or extracellular vesicles to the CNS environment [18,19]. Therapeutic approaches utilizing stem cells have also shown that they could affect both innate and adaptive immune response and regulate inflammation when administered intrathecally in ALS patients [20]. The lineage-negative stem/progenitor cells (Lin^–^) population is a heterogenous fraction of cells, easily obtained from bone marrow (BM) or umbilical cord blood (UCB), consisting of cells which do not express markers characteristic of mature hematopoietic lineages but that in turn are enriched in surface markers involved in the migration, adhesion, and homing of hematopoietic stem cells to the bone marrow (CD164, CD162, and CXCR4) [21]. Moreover, they have been shown to express higher levels of NTs when compared to other nucleated UCB cells, and have proved to be a feasible and safe means of trophic support provision when administered via a lumbar puncture [22,23]. Furthermore, in previous studies of our research group we have observed the modulation of immune response after a single, intrathecal administration of Lin^–^ cells in ALS subjects [24]. Therefore, this cell population constitutes a promising tool aimed at both providing a trophic support for degenerating motor neurons in CNS and acting to attenuate the activation of immune response. The aim of this study was to investigate the effects of the repeated, intrathecal administration of BM-derived Lin^–^ cells on molecular pathways on a local and systemic level, with particular emphasis on processes involved in the regulation of immune response.

## 2. Materials and Methods

### 2.1. Study Design

The study was designed as a prospective, open-label, and nonrandomized clinical trial. A control, placebo group was not included as it raised ethical doubts and would also pose substantial difficulties in matching subjects in the studied and control groups due to the differences in disease course and duration and the vast heterogeneity among ALS patients. Before the enrollment, the patients underwent a natural history observation to correctly estimate their life expectancy. Autologous Lin^–^ cell administration was repeated three times in six-week time intervals. Each application was followed by 7 days of hospitalization. The patients were also asked to attend the follow-up visit on the 28th day after each procedure.

### 2.2. Patients

Forty-five sALS patients (21 female and 24 male) of Polish origin between 27 and 65 years old (53.8 ± 9.35) were enrolled in the study after it obtained the approval of the local ethics committee of the Pomeranian Medical University in Szczecin (approval code: KB-00012/06/10). ALS was diagnosed based on the revised El Escorial Criteria [25,26]. All the patients gave informed consent before the enrollment. The disease progression measured using the revised ALS Functional Rating Scale (ALS-FRSr) assessment was mild to moderate, with satisfactory spinal and bulbar motor functions, and the forced vital capacity (FVC) was greater than or equal to 50%. The presence of comorbidities, an age of above 65, and any medication which could affect bone marrow function were included in the exclusion criteria. In general, as the study design implied triple cell administration and a consecutive follow-up period, the minimum predicted survival time was at least 12 months. All the patients had to meet observation criteria of riluzole-controlled disease for 3 months preceding the use of cell therapy. The study was registered as a clinical trial (number: NCT02193893) and performed in accordance with the Declaration of Helsinki.

### 2.3. Neurological Assessment

The patients’ functional performance was assessed using two questionnaire-based scales: ALS-FRSr and the Norris scale. ALS-FRSr is based on a questionnaire which addresses everyday functions divided into four clinical domains (bulbar function, fine motor function, gross motor function, respiratory parts), which allow for the assessment of limb, bulbar, respiratory, and trunk impairments and disabilities [27]. All the patients were examined using both scales before the injection of Lin^–^ cells, and on the 3rd, 5th, 7th, and 28th day after each procedure of cell administration.

### 2.4. Bone Marrow Collection and Isolation of Lin^–^ Cells

Each time, prior to the collection of BM the patients gave their informed consent for the procedure. The collection site (posterior iliac crest) was locally anesthetized. The volume of collected BM together with anticoagulant solution ranged from 44 to 156 mL (122 ± 22.71). All the subsequent isolation steps were performed in a closed isolator system Xvivo (BioSpherix, Ltd., Parish, NY, USA). Firstly, the mononucleated cell suspension was obtained via centrifuging in Lymphocyte Separation Medium (MP Biomedicals, Santa Ana, CA, USA), and then the immunomagnetic negative isolation of Lin^–^ cells was performed with a commercially available kit according to the manufacturer’s protocol (Miltenyi Biotec, Auburn, AL, USA), as described previously [28]. Isolated cells were then suspended in 2 mL of sterile phosphate-buffered saline (PBS, Polfa, Kutno, Poland). The number of obtained cells differed between patients and ranged from 0.44 to 20.5 × 10^6^ (mean 7.3 × 10^6^ ± 6.3).

### 2.5. Lineage-Negative Cells Injection

All the isolated Lin^–^ cells were administered intrathecally into subarachnoid space. The lumbar puncture was performed between the L3/L4 or L4/L5 vertebrae. To avoid a potential post dural puncture headache (PDPH), the patients were recommended to remain in a supine position for at least 24 h.

### 2.6. Multiplex Luminex Assay

To assess the concentrations of CRP, TNF-α, and its receptor—tumor necrosis factor receptor 1 (TNF-R1)—blood collected using ethylenediaminetetraacetic acid (EDTA) as an anticoagulating factor and CSF samples were collected before each of three applications of Lin^–^ cells and 7 days later. Both types of samples were first centrifuged (10 min, 2000 rpm) to remove the cellular fraction. The plasma and CSF samples were then stored in −80 °C until the Luminex (Luminex Corporation, Austin, TX, USA) assays were performed. The analysis was performed according to manuals provided with the multiplex reagent kit (Human Magnetic Luminex Assay, R&D Systems, Minneapolis, MN, USA) using the Luminex 200 analyzer. The final concentration was calculated based on the averaged readings from the duplicates for each sample.

### 2.7. miRNA Expression Analysis

The expression of selected miRNAs (miR-155-5p, miR-378a-5p, miR-206-5p, and miR-1-5p) was assessed using the qPCR technique in samples of plasma and CSF collected before each injection of Lin^–^ cells and one week later. The isolation of circulating miRNA was performed using the commercially available NucleoSpin miRNA Plasma kit (Macherey-Nagel, Düren, Germany) according to the manufacturer’s protocol. Synthetic *Caenorhabditis elegans* miRNA-39 was introduced to each sample prior to the isolation as an internal control. Afterwards, the samples were stored in −80 °C until the further steps of the analysis. The reverse transcription of miRNA isolated from both collected bodily fluids was performed using the qScript microRNA cDNA Synthesis Kit (QuantaBio, Beverly, MA, USA). An amount of 4 µL of miRNA were used for each reverse transcription reaction. The Bio-Rad CFX 96 system (Bio-Rad, Hercules, CA, USA) was used to perform the qPCR reaction. The reaction solution consisted of 1 µL of cDNA, 5 µL of iQ SYBR Green Supermix (Bio-Rad, Hercules, CA, USA), Universal Primer provided with a reverse transcription kit, and a forward primer specific to each of the analyzed miRNAs (for miR-155-5p—5′ CGCAGTTAATGCTAATCGTGATAG, for miR-378a-5p—5′ GCTCCTGACTCCAGGTC, for miR-1-5p—5′ CGCAGACATACTTCTTTATATGC, for miR-206-5p—5′ CAGTGGAATGTAAGGAAGTGTG and for c-miR-39—5′ GTCACCGGGTGTAAATCAG). As a negative control, samples of miRNA prior to the reverse transcription reaction were used. The total reaction volume was 10 µL, and for each sample there were two technical replicates. The qPCR reaction was performed as follows: 95 °C for 5 min; 40 cycles of denaturation (95 °C, 20 s), annealing (62 °C, 30 s), and elongation (70 °C, 30 s); followed by an increase from 65 °C to 95 °C to assess the melting temperatures. A melt curve analysis was performed to test whether the amplification reaction was specific. The mean CT values with SD for selected miRNAs were as follows: miR-155-5p—26.67 ± 3.14 and 23.08 ± 7.86; miR-378a-5p—27.03 ± 4.31 and 26.49 ± 6.52; miR-206-5p—29.23 ± 3.88 and 28.63 ± 4.29; miR1-5p—28.78 ± 2.12 and 27.92 ± 2.67; and for c-miR-39 21.06 ± 1.48 and 21.32 ± 1.87, for CSF and plasma, respectively. CT 35 was considered as a detection cut-off point. The relative expression was calculated in relation to the spiked-in synthetic *C. elegans* miR-39 as 2^−ΔΔ*C*T^.

### 2.8. Gene Chip Microarray

Blood samples for a gene chip microarray analysis were obtained from three patients on the day of the BM collection/cell injection (day 0) and on the 7th day after the Lin^–^ cell administration. We have selected patients’ samples for analysis based on a retrospective analysis of the changes in their functional outcomes after each injection of Lin^–^ cells.

Total RNA was isolated from the collected samples using the PARIS Kit (Thermo Fisher Scientific, Waltham, MA, USA). Microarrays were prepared as described previously [24]. The CEL files were analyzed using the BioConductor software with the “affy” and “oligo” packages installed for background correction and biological processes annotation, respectively. The obtained data were analyzed using the K-means clustering approach, which allows for the identification of subgroups of co-regulated differentially expressed genes (DEGs). The number of groups was calculated using the sum of squared errors estimation (SSE). Afterwards, the data have been scaled and centered and subjected to K-means clustering, as described in Baumert et al. [24].

### 2.9. Statistical Analysis

The distribution of obtained data was tested using the Shapiro–Wilk test. The differences between certain analyzed timepoints were assessed using the Wilcoxon signed-rank test. Spearman’s rank correlation coefficient was used to present the relationships between different tested parameters. To compare the differences between two analyzed groups (responders vs. non-responders), the Mann-Whitney U test was applied. *p* values smaller than or equal to 0.05 were considered to indicate statistical significance. All the statistical analyses were performed with STATISTICA 12.5 PL.

## 3. Results

In the experiment design, there were three consecutive applications of autologous cells isolated from the bone marrow of sALS patients, each planned six-week apart. Two women did not complete the study (they resigned due to personal reasons); the rest of the patients remained in the experiment for all three cell injections. However, three patients did not appear for follow-up examinations. Therefore, final data are presented for a total of 40 patients.

**Figure 1:**
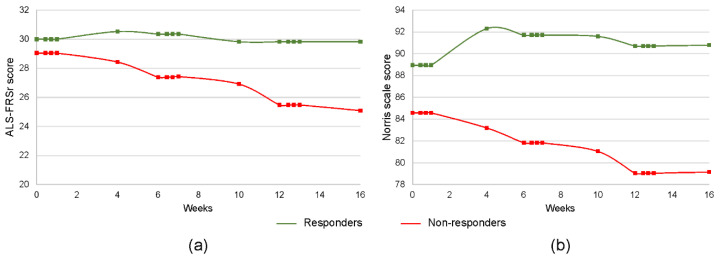
Results of the ALS-FRSr (**a**) and Norris scale (**b**) assessments for two groups: responders (green, n = 17) and non-responders (red, n = 23).

Based on the retrospective assessment of the functional response to the cell injections, we have divided the patients into two groups: (i) responders (n = 17) and (ii) non-responders (n = 23). The ALS-FRSr was chosen as an indicator of clinical response to the cell administration, as it has been previously described that this scale correlates best with the predicted survival time [29]. In the responders group, we have included those patients whose condition did not deteriorate by more than 1 point in ALS-FRSr between day 0 before the first administration of Lin^–^ cells and the final functional assessment on the 28th day after the third cell injection. All the patients whose results of the ALS-FRSr assessment at the end of the experiment worsened by 2 or more points were included in the non-responders group. The general characteristic of both groups is presented in Table 1. The changes in results of the ALS-FRSr and Norris scale assessment during the observation period have been summarized in Figure 1. There were no statistically significant differences between the groups concerning age, sex, disease duration and onset, the number of cells administered, and the initial neurological condition. A correlation analysis revealed statistically significant negative relationships between the patients’ ages and disease durations (Spearman’s correlation coefficient—r_s_ = −0.43) and between their age and the number of isolated Lin^–^ cells (r_s_ = −0.42).

### 3.1. Concentration of Inflammatory Cytokines

**Figure 2:**
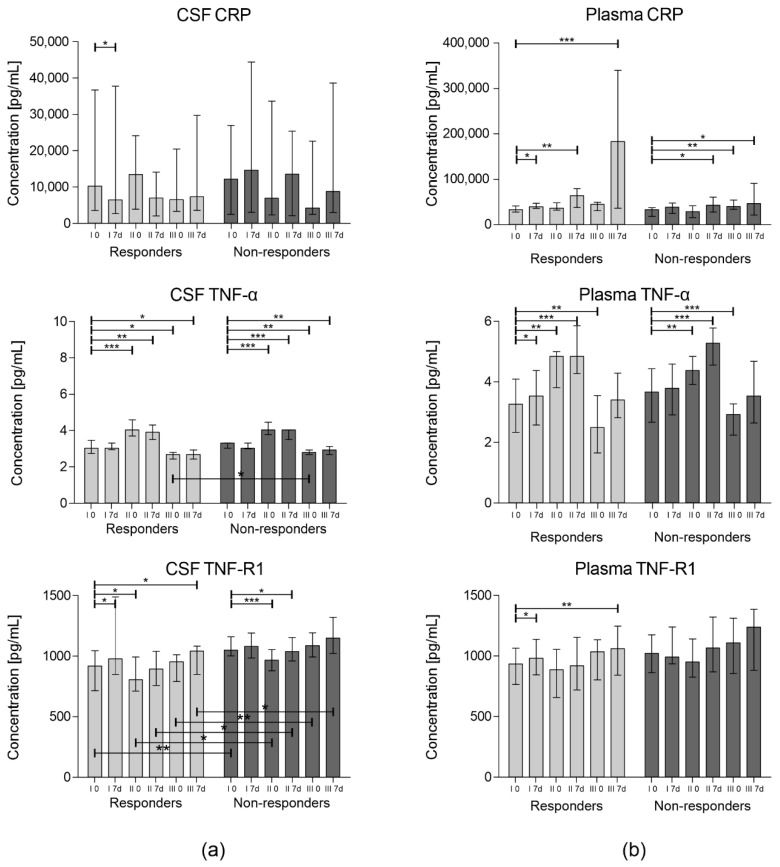
Concentration (pg/mL) of analyzed cytokines in the bodily fluids of ALS patients before (0) and one week after (7 d) each of the cell administrations (I, II, and III): (**a**) cerebrospinal fluid (CSF) concentrations; (**b**) concentrations in plasma. Data are presented as the median value with the interquartile range. Statistical testing between different timepoints in each of the analyzed groups (responders and non-responders) was performed using the Wilcoxon signed-rank test. To compare certain timepoints between the groups, the Mann–Whitney U test was applied. Level of significance—* *p* < 0.05, ** *p* < 0.01, *** *p* < 0.001. CRP: C-reactive protein; TNF-α: tumor necrosis factor α; TNF-R1—tumor necrosis factor receptor 1.

The concentrations of CRP, TNF-α, and TNF-R1 were measured in the CSF and plasma in different time-points using the multiplex Luminex technique. The results of the analyses are presented in Figure 2.

It is worth noticing that the subjects from the responders group demonstrated a statistically significant decrease in the CRP level in CSF on the 7th day post first Lin^–^ cell administration (*p* = 0.02). In parallel, a significant difference between day 7 after the first procedure and day 0 (baseline) between the groups was confirmed (*p* = 0.02). There was a decrease in the responders and an increase in the non-responders groups, respectively. The concentration of TNF-R1 in the CSF in almost all time-points (except from day 7 following first application) was statistically higher in the non-responders group. On the 7th day after each procedure, increased concentrations of TNF-R1 in the CSF and TNF-α in plasma compared to day 0 in both groups were observed.

### 3.2. miRNA Expression

**Figure 3:**
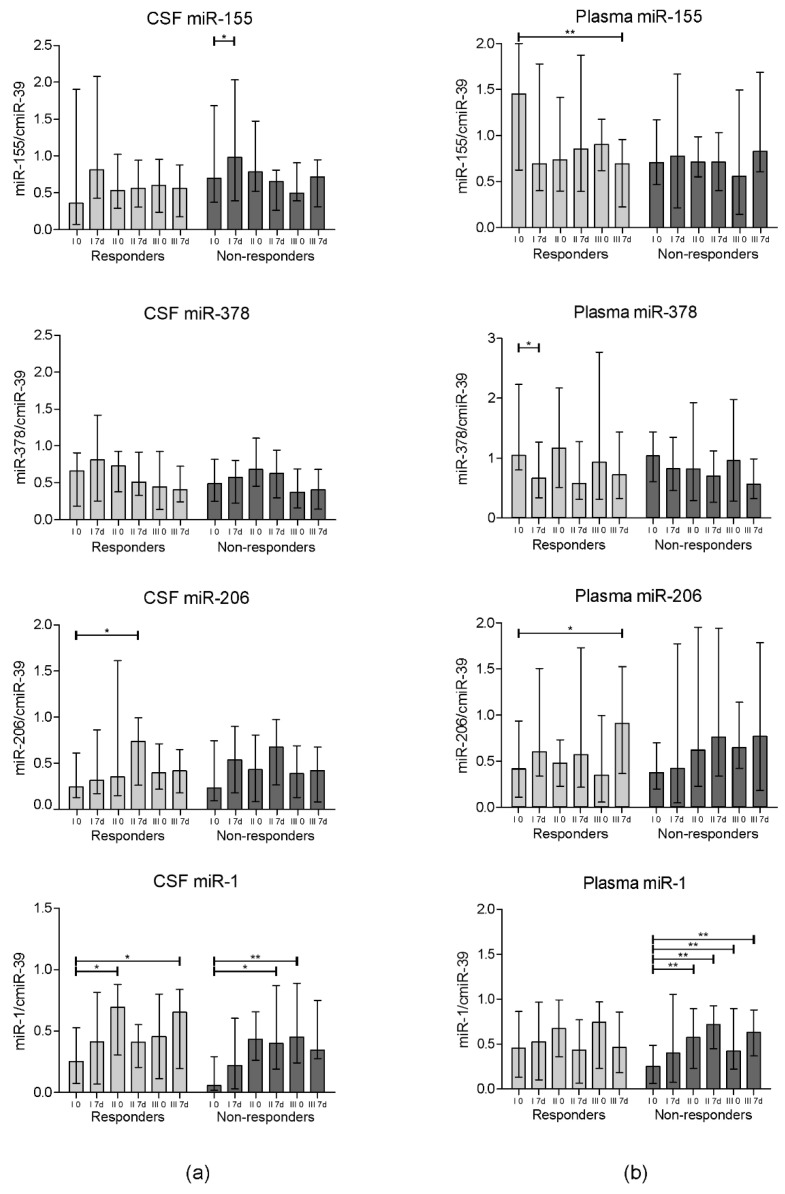
Relative expression of miR-155, miR-378, miR-206, and miR-1 in the bodily fluids of ALS patients before (0) and one week after (7 d) each of the cell administrations (I, II, and III) in: (**a**) CSF; (**b**) plasma. Data are presented as the median value with the interquartile range. Statistical testing between different timepoints in each of the analyzed groups (responders and non-responders) was performed using the Wilcoxon signed-rank test. To compare certain timepoints between the groups, the Mann–Whitney U test was applied. Level of significance—* *p* < 0.05, ** *p* < 0.01.

The miRNA expression in the collected CSF and plasma samples was assessed on the day of the cell administration and 7 days after each of three procedures using the qPCR technique. As the amount of circulating miRNA in bodily fluids is very small, to normalize the data the synthetic miRNA was introduced to the sample prior to the miRNA isolation. We have analyzed the concentrations of four miRNAs in which correlations with the ALS course and progression have been previously suggested [30,31,32]: miR-155, miR-378, miR-206, and miR-1. The expression analysis results have been presented in Figure 3. Interestingly, the expression of miR-155 notably increased in CSF on the 7th day after the first cell injection in the non-responders’ group (*p* = 0.04). In contrast, a significantly lower miR-155 expression in plasma was observed in the responders’ group on day 7 after the final cell administration in comparison to the baseline (*p* = 0.007). A statistically significant decrease in the miR-378 expression in plasma in the responders’ group was confirmed on day 7 after the first cell application (*p* = 0.01). We have observed a significant increase in the expression of miR-206 in the responders group in both analyzed bodily fluids (*p* = 0.02 for CSF on the 7th day after the second procedure and *p* = 0.04 in plasma on the 7th day after the third procedure) compared to the baseline. No significant differences were observed in the corresponding timepoints between groups.

### 3.3. Global Gene Expression

**Figure 4:**
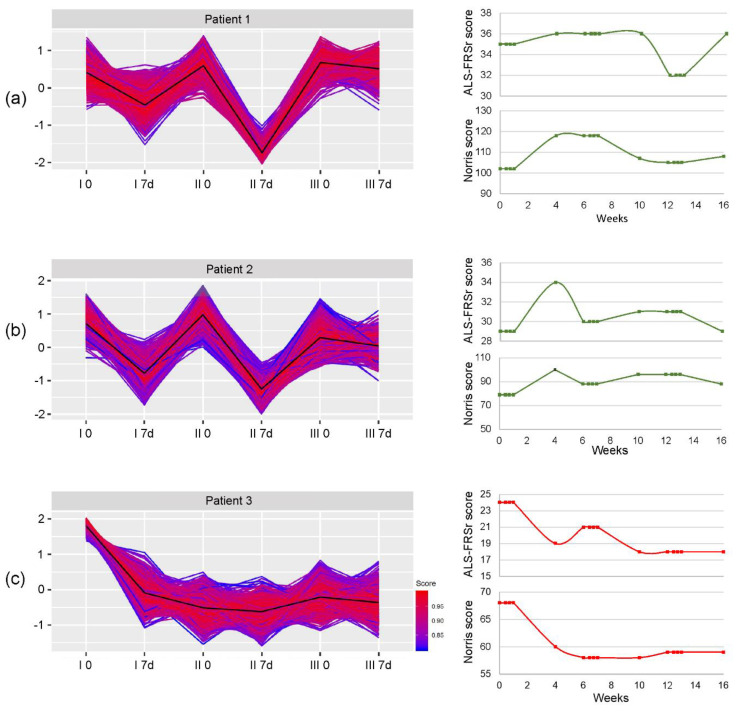
Changes in the expression of genes assigned to biological processes related to neutrophil activation, degranulation, and neutrophil-mediated immunity in three patients: (**a**) Patient 1, assigned to the responders group; (**b**) Patient 2, also assigned to the responders group; (**c**) Patient 3, who has been assigned to the non-responders group. Additionally, the results of functional assessment with the ALS-FRSr and Norris scale have been presented for each patient separately. Green curve—patients qualifying for the responders group; red curve—patients representing the non-responders group.

**Figure 5:**
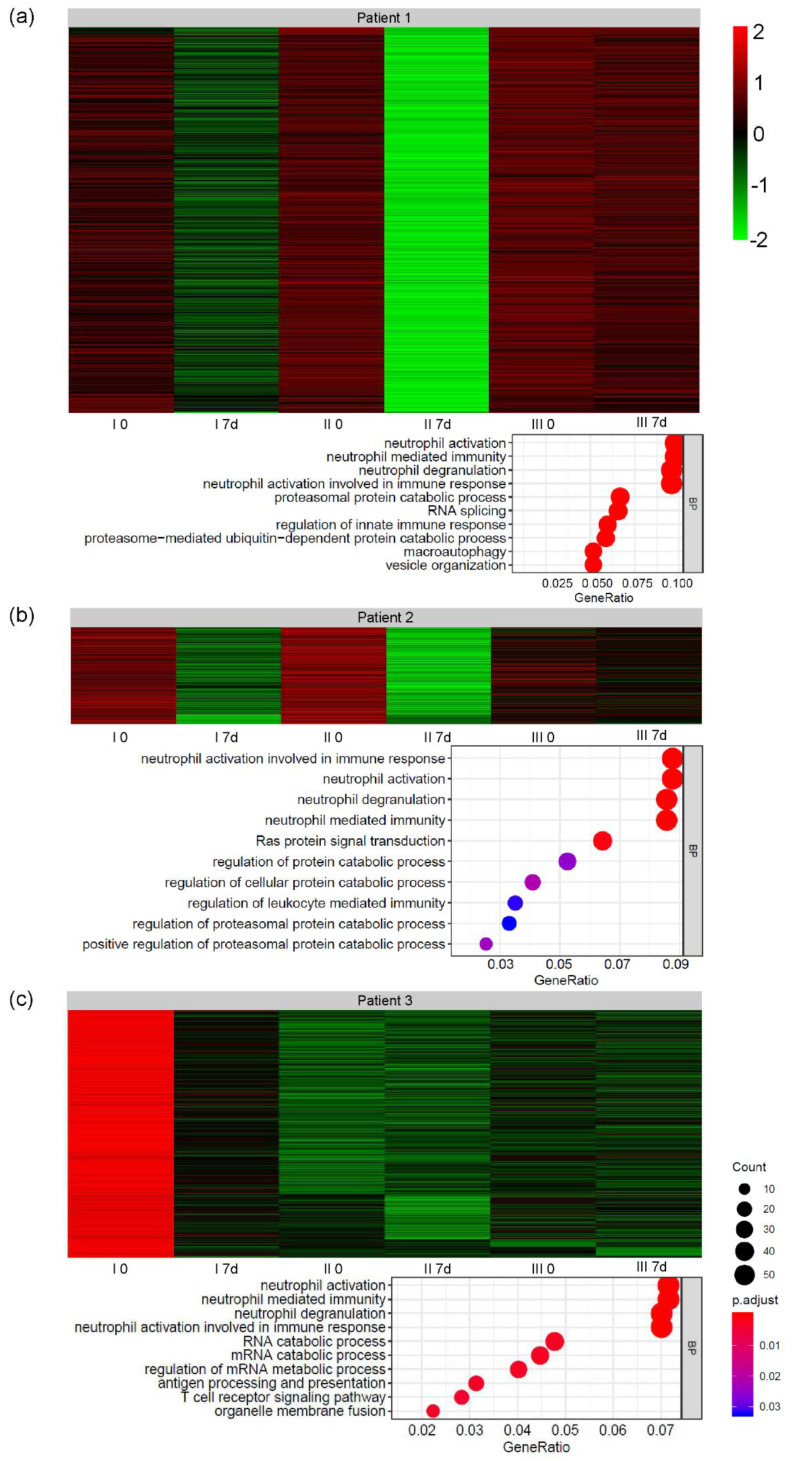
Heatmaps representing the expression of genes assigned to the neutrophil-related gene cluster in all analyzed timepoints for 3 patients: (**a**) Patient 1, (**b**) Patient 2, and (**c**) Patient 3. Bubble plots depict the assignment of genes to biological processes based on gene ontology (GO).

Gene microarrays have been implemented to assess global shifts in the expression of genes involved in the regulation of immune system. The K-clustering approach has distinguished five clusters of significantly changed genes in three analyzed representative patients. Two patients included in the gene microarray analysis were assigned to the responders group (Patient 1 and Patient 2), while one represented the group of non-responders (Patient 3). The assignment of differentially expressed genes to relevant gene ontology terms revealed that one cluster containing genes responsible for the activation and regulation of neutrophil activation, neutrophil activation involved in immune response, neutrophil-mediated immunity, and neutrophil degranulation was repeated in all the analyzed samples. The changes in the expression of genes contained in this cluster for each patient individually have been presented in Figure 4. Additionally, genes from the described cluster have been enlisted as a Supplementary Table S1 for each patient separately. In Figure 5, we have also presented heatmaps representing the expression changes in differentially expressed genes from the abovementioned cluster in all analyzed time-points and the assignment of those genes to biological processes based on their gene ontology (GO). We have observed a general decrease in the expression of genes assigned to analyzed cluster in all three patients; however, in patients who responded to the therapy the decrease repeated after each cell administration. In Patient 3, who represented the non-responders group, the decrease after the second and third injection of the cells was not as pronounced.

## 4. Discussion

The main goal of the presented study was to assess the effect of repeated intrathecal Lin^–^ cell administration on the regulation of immunological pathways. However, it is also the first report in which the results of a clinical trial involving triple intrathecal Lin^–^ cell injections in sALS patients have been presented. It is crucial to note that the study found no significant clinical adverse effects of such procedures, which further confirms that bone marrow constitutes a feasible source of this cell population for intrathecal transplant procedures. Moreover, we have established that a certain group of patients responded to the Lin^–^ administration in such a way that their functional condition improved. In our previous studies [23,24,33], we demonstrated that the beneficial effect of Lin^–^ cell therapy was mainly due to trophic support via the secretion of neurotrophins and pro-angiogenic factors, whose peak was observed after 3–5 days and corresponded with the best clinical effect up to the 7th day. In the current study, the clinical improvement was not rapid nor short-term, but rather prolonged in time, showing the best results at about 4 to 8 weeks post cell application according to ALS-FRS and after up to 16 weeks according to the Norris scale (Figure 1). The observation of different modulations of immune mechanisms in the responders and non-responders indirectly proves that the obtained clinical effect was achieved not only by the paracrine effect, but also by the modulation of the immunological pathways. Based on this observation, we aimed to sustain this immunomodulatory effect via the repeated intrathecal administration of Lin^–^ cells.

We have assessed the influence of Lin^–^ cell injection on two levels: locally in the CSF, and systemically in the blood plasma. CRP is an acute-phase inflammatory protein synthesized by hepatocytes, smooth muscle cells, adipocytes, macrophages, and lymphocytes [33]. The transcription of *CRP* gene enhances in response to interleukin-6 (IL-6) stimulation, however it is altered also by other factors, including age and cigarette smoking [34]. In this study, we have not observed any significant correlations between the abovementioned parameters and the CRP concentration in CSF, nor in plasma. The obtained results revealed a statistically significant decrease in the concentration of CRP in the CSF of patients assigned to the responders group, while in the case of the non-responders the concentrations shifted in an opposite direction one week after the administration of Lin^–^ cells. Again, after the second cell administration we have observed a similar tendency, however it was not significant. We hypothesize that, as the levels of this protein in central nervous system (CNS) environment reflect the ongoing inflammatory processes, those are inhibited in the responders after the injection of Lin^–^ cells. A high baseline CRP level in the CSF and its further elevation after Lin^–^ cell administration seems to be an adverse prognostic factor announcing rapid disease progression and the inability to control its course. On the contrary, a lower baseline CRP level in the CSF and its further reduction on the 7th day after the procedure might predict responsiveness to applied therapy and substantial benefits for ALS patients. These findings remain in harmony with our preliminary results presented in a previous report [24], where the CRP levels in CSF were negatively correlated with the clinical outcomes of ALS patients. The CRP serum levels have been previously proposed as a marker indicating the more rapid progression of ALS [12]. Indeed, the concentrations of this protein increased gradually in the analyzed group. Interestingly, we observed a significant, very pronounced increase in the CRP level in plasma after the third administration of Lin^–^ cells in the responders group, which in this case does not correspond with worse clinical outcomes. This issue needs further investigation to precisely assess the underlying molecular mechanisms of this process. The second of the analyzed proteins was TNF-α. This protein is a potent mediator of inflammation. It is expressed by activated monocytes; macrophages; and non-immune cells, including endothelial cells and fibroblasts [35]. It is also secreted by the cells of the CNS: microglia; astrocytes; ependymal cells; and, in case of cerebral ischemia, by injured neurons [36]. TNF-α exerts its pro-apoptotic actions via interaction with TNF-R1 [37]. Physiologically, TNF-R1-mediated cell death is triggered by intracellular aberrations (cell cycle inhibition, improper protein synthesis) to activate caspase cascades and eventually eliminate those improperly functioning cells [38]. However, in neurodegenerative diseases, where the chronic neuroinflammation leads to a permanent increase in the concentration of TNF-α, the actions of this protein result in damage to oligodendrocytes, enhanced demyelination, and the disruption of the BBB [39,40]. Additionally, in ALS increased levels of circulating TNF-α are also considered as a factor contributing to muscle wasting [41]. The inhibition of TNF-α has been proposed as one of the possible routes to attenuate chronic neuroinflammation in the CNS of patients with ALS and other neurodegenerative diseases [42]. In our study, we observed in general low levels of this protein in both examined bodily fluids. The measured concentrations rose after intervention both locally and systemically, but the change was more pronounced in blood plasma. Comparing the TNF-α level, no significant differences between the two analyzed groups in corresponding timepoints were observed, except in CSF on day 0 of the third application. Considering the receptor for TNF-α–TNF-R1, a membrane receptor which has also been reported to circulate in a soluble form in plasma and CSF, its levels also increased one week after the administration of Lin^–^ cells, however the concentrations were significantly lower in the responders than in the non-responders group in five out of six analyzed time-points. In this point of discussion, it should be also noted that TNF-α may exert its functions also by interaction with other receptors, which may lead to completely opposing effects. There is also evidence supporting the thesis that TNF-R1 may also exert neuroprotective actions, such as the sensitization of neurons to erythropoietin and vascular endothelial growth factor (VEGF) following ischemic episodes [43], or the stimulation of glial-derived neurotrophic factor (GDNF) release [44].

The second group of factors involved in the regulation of immunological pathways which we have investigated were microRNAs (miRNAs). Those small, non-coding RNA fragments take part in the regulation of gene expression on the translational and post-translational level. The search for markers of various diseases among miRNAs has continued since their first discovery in 1993, and it has brought promising results, especially in certain types of cancers/tumors [45]. In our research, we have focused on the expression of four miRNAs which have been previously mentioned as related to the progression of ALS: miR-155 and miR-378, described as “immune-miRs” due to their involvement in the regulation of genes related to immune response; and miR-206 and miR-1, included in the group of so-called “myo-miRs” due to their effects on the proliferation of muscle cells [46]. miR-155 has been previously described as one involved in the promotion of pro-inflammatory pathways via the repression of Src homology-2 domain-containing inositol 5 phosphatase 1 (SHIP1) and as a suppressor of cytokine signaling-1 [47,48]. It has been also shown to promote neuroinflammation [49]. Moreover, Koval et al. have reported that this molecule is overexpressed in the spinal cords of ALS patients and in SOD1^G93A^ mice, and proposed miR-155 not only as a marker of the disease but also as a potential therapeutic target [50]. In our study, we have observed a significant increase in the expression level of this molecule week after the first administration of cells in the CSF of the non-responders group. The initial CSF level was also higher in this group when compared to the responders, which may constitute a marker of prognosed response to the intervention. In plasma, there was a significant decrease in miR-155 expression in the responders group one week after the first cell injection, which suggests a decrease in the activation of systemic inflammatory pathways. Next, the analyzed “immune-miR” was miR-378, which has been described as one correlated with muscle cachexia and lipolysis, which are one of the hallmarks of ALS [51,52]. Our results have revealed an interesting pattern of the expression of miR-378 in plasma—its level was decreased one week after each of the procedures. Those changes were higher in the responders group. However, only the change in the responders group after the first injection of Lin^–^ cells was statistically significant, and the overall trend is clearly visible. The analysis of miR-378 in CSF again showed a gradual decrease in expression in consecutive time-points. miR-206 is included in the group of “myo-miRs”, as it is one of the regulators in neuromuscular signaling. In the study on the mice ALS model (SOD1^G93A^), Williams et al. confirmed that the loss of miR-206 led to increased disease progression, expressed by accelerated muscle atrophy, and that it promotes the formation of new neuromuscular junctions (NMJs) [53]. The expression pattern of this miRNA in the presented study was similar in both the described groups of patients, however the increase in its expression was significant in the responders group only. The last of the assessed miRNAs was miR-1. This molecule was chosen for analysis due to its previously described involvement in the regeneration and differentiation of muscle cells [54]. It has been also proposed that the injection of this molecule into muscle in a rat injury model induced the expression of myogenic markers, thus stimulating the regeneration of muscle fibers [55]. Therefore, we were curious to assess whether the expression level of miR-1 would correspond with the disease progression and muscle weakening in the ALS patients enrolled in the study. The obtained results, however, did not show a correlation with clinical outcome, as the expression increased in the CSF as well as in plasma in both study groups.

The last step of our study was the analysis of global gene expression. Using a clustering approach, we were able to see groups of genes which were significantly changed in the assessed time-points. The analysis revealed five cluster of differentially expressed genes (DEGs) in all the patients included in the analysis. One of the clusters was comprised of genes assigned to corresponding processes involved in the regulation of the neutrophil-mediated immune response. Therefore, we have decided to present changes in the expression of this cluster in relation to patients’ clinical outcomes. We have observed that the expression of genes regulating the activation of neutrophils decreased one week after the procedure of Lin^–^ cell administration. This decrease was most significant in the patient who responded best to the intervention. In the patient representing the non-responders’ group, there was also a prominent decrease in the expression of this cluster of genes, however the changes between subsequent time-points were not as pronounced as in the individuals included in the responders group. The number of DEGs in this patient was also distinctly lower (as shown in Supplementary Table S1). This decrease indicates the influence of cells’ injection on the downregulation of systemic inflammation. Moreover, this influence on neutrophil activation may exert beneficial clinical effects, as in animal studies it has been shown that these cells play a pivotal role in motor nerve demyelination and the loss of muscle fibers [56].

Overall, almost half of the study group (responders, n = 17) remained stable or improved in clinical outcome during the experiment duration (4 months). Taking into consideration the typical rapid progression of ALS and the mean survival time of 3–5 years after disease onset, this time period without worsening constitutes a definitely positive outcome. Our study proves that the intrathecal therapy utilizing autologous bone marrow-derived Lin^–^ cells influences the innate and adaptive immune response, especially in patients responding well to the intervention, on both a local and systemic level. The observed effect of SPC applications may result from the paracrine action of a number of factors produced by stem/progenitor cells.

Despite our best efforts, the study had some limitations which we would like to note. Firstly, the study design did not include a control placebo group. This issue has been widely discussed inside our research group and has raised many ethical remarks. Therefore, with full awareness of the consequences, we have decided to administer cells to all the recruited sALS patients. Secondly, recent reports have shown numerous inborn stem cell defects in ALS that may limit the beneficial effects of cell therapies or even render them ineffective [57,58]. In our study, we did not perform tests in this respect. Thirdly, taking into consideration the high variability of miRNAs between patients, the full miRNA mapping instead of the evaluation of only selected immune-related miRNAs would be of much greater clinical importance. Finally, the time points of clinical assessment and sample collection, except for the first hospitalization period (7 days after each procedure), were sparse, which resulted from the fact that the patients were geographically distanced from our research center and were not able to show up more frequently for check-up examinations. Given the above, we believe that our study still provides new insights into the mechanisms of response to Lin^–^ cells therapy and casts a new light on its influence on the regulation of immune pathways in ALS patients.

## Figures and Tables

**Table 1:** Patients’ characteristics. Responders—patients whose functional condition assessed using amyotrophic lateral sclerosis (ALS) Functional Rating Scale (ALS-FRSr) did not deteriorate by more than 1 point during the observation period. Non-responders—patients whose results in ALS-FRSr were lower on the 28th day after the last administration of Lin^–^ cells by 2 or more points than before the first procedure.

		Responders (n = 17)	Non-Responders (n = 23)	*p*-Value
Age (mean ± SD, years)		53.1 ± 9.5	54.4 ± 8.8	0.76
Gender (Male/Female)		8/9	14/9	0.52
Disease duration (mean ± SD, months)		27.9 ± 20.2	28.9 ± 29.7	0.29
Disease onset (bulbar/limb)		5/12	5/18	0.72
Number of administered Lin^–^ cells (mean ± SD)	I	8.1 × 10^6^ ± 6.3	5.5 × 10^6^ ± 6.0	0.17
	II	7.9 × 10^6^ ± 6.0	6.7 × 10^6^ ± 6.8	0.24
	III	7.5 × 10^6^ ± 9.8	7.0 × 10^6^ ± 5.0	0.48
Baseline ALS-FRSr (mean ± SD, points)		30.1 ± 4.3	29 ± 5.6	0.39
Baseline Norris scale (mean ± SD, points)		90.2 ± 16.2	79.4 ± 14.3	0.25

## Supplementary Materials

The following are available online at https://www.mdpi.com/2073-4409/9/8/1822/s1: Table S1: Differentially expressed genes assigned to the presented gene cluster for each patient individually.
